# Does auxiliary cerclage wiring provide intrinsic stability in cephalomedullary nailing of trochanteric and subtrochanteric fractures?

**DOI:** 10.1007/s00264-020-04795-4

**Published:** 2020-09-12

**Authors:** Julia Rehme, Alexander Woltmann, Andreas Brand, Christian von Rüden

**Affiliations:** 1 Department of Trauma Surgery, BG Unfallklinik Murnau, Professor Küntscher Str. 8, 82418 Murnau, Germany; 2 Institute for Biomechanics, BG Unfallklinik Murnau, Murnau, Germany; 3 Institute for Biomechanics, Paracelsus Medical University, Salzburg, Austria

**Keywords:** Trochanteric fracture, Cephalomedullary nail, Cable cerclage wiring, LEFS, RUSH, Intrinsic stability, Long-term outcome

## Abstract

**Purpose:**

The aim of this study was to assess functional and radiological results following cephalomedullary nailing with and without use of auxiliary cable cerclages in a large series of trochanteric and subtrochanteric femoral fractures.

**Methods:**

In a retrospective study of prospectively collected data between January 2014 and March 2019, a total of 260 consecutive patients (155 women and 105 men) with the diagnosis of AO/OTA A1 to A3 fractures were included. The mean age of patients was 76.4 ± 15.6 years. According to the AO/OTA classification, 72 A1 fractures, 124 A2 fractures, and 64 A3 fractures were found. In 72 patients with auxiliary cerclage wiring three A1 fractures, 27 A2 fractures and 42 A3 fractures were assessed. In the patient group with auxiliary cerclages, fracture healing according to the Radiographic Union Score for Hip (RUSH) within one year after surgery was assessed in 68 out of 72 patients (healing rate 94%). The mean RUSH in the group with cerclages was 28.7 ± 2.2 points and was 28.5 ± 2.2 points in the group without cerclages (*p* = 0.72). In 91 patients available for a complete follow-up, mean functional outcome according to the Lower Extremity Functional Scale (LEFS) was 65.3 ± 17.2 points in the group with cerclages versus 58.4 ± 21 points in the group without cerclages (*p* = 0.04).

**Conclusion:**

The additional use of cerclages provides intrinsic stability and enables axial alignment and medial cortical support during anatomical fracture reduction and cephalomedullary nail insertion. In the current study, this technique resulted in significantly better functional long-term outcomes than without cerclages. Therefore, it can be recommended as a useful supportive tool especially in comminuted trochanteric and subtrochanteric fractures. Trial registration number DRKS00020550, 01/30/2020, retrospectively registered.

## Introduction

The proximal femoral fracture is a typical injury to the elderly [1]. A fracture can be demonstrated with increasing age, especially following minor trauma [2, 3]. Due to the demographic change, an increasing incidence of these fractures may be expected in the future. Basically, trochanteric fractures include two-fragment Arbeitsgemeinschaft für Osteosynthesefragen (AO)/Orthopaedic Trauma Association (OTA) type 31 A1 fractures, most frequently multi-fragmentary trochanteric AO/OTA type A2 fractures, and rarely reversed AO/OTA type A3 fractures and can be distinguished from highly unstable subtrochanteric fractures [4, 5]. A general consensus on the surgical management of these unstable fractures remains controversial. Cephalomedullary nailing currently is the gold standard for internal stabilization [6]. The extent contributes to the therapy decision as well as to the direction of instability, which increases from proximal to distal. A distinction can be made between the mediolateral, the rotatory, and the craniocaudal instability. The aim of internal fixation is to neutralize this instability biomechanically through the inserted implant in such a way that the anatomical fracture reduction and retention is feasible. While extramedullary implants are recommended for stable fractures, intramedullary force carriers will be favoured with increasing rotational instability or mediolateral component (AO/OTA type A2 fractures) [7]. With additional craniocaudal instability and shaft extensions, extended cephalomedullary nails are used as standard implants [7]. For fracture healing, anatomical reduction, correct choice of implant, and exact positioning of the lag screw are essential preconditions for primary stability [8, 9]. Furthermore, the primary stability of an implant is given when bending forces arising from normal loads and the resulting displacements during implant insertion do not weaken, damage, or inhibit the mechanical osseous integration and do not have any negative effects on the biological osseous integration [7, 10]. Due to the fracture configuration, there is often an indication for the use of one or more supplemental cable cerclages aiming for safe fracture reduction as well as increased intrinsic primary stability as a prerequisite for complication-free osseous healing. Basically, adequate primary stability is always a precondition for timely secondary stability and osseous integration [11]. However, there is still disagreement whether auxiliary cerclages may only be used temporarily as a reduction aid or whether they can be left in situ [12–14]. The use of cerclages is also suspected of disrupting the blood flow to the periosteum depending on the position and thus endangering fracture healing [15–18]. Cerclages also may harbor the risk of vascular and nervous complications in terms of the nature of the system [19–21].

Data on clinical and radiological long-term course in a large number of patients has been scarce so far in the literature. Therefore, the aim of this study was to evaluate long-term functional and radiological results following cephalomedullary nailing with and without the use of auxiliary cable cerclages in a large series of trochanteric and subtrochanteric femoral fractures.

## Methods

**Fig. 1:**
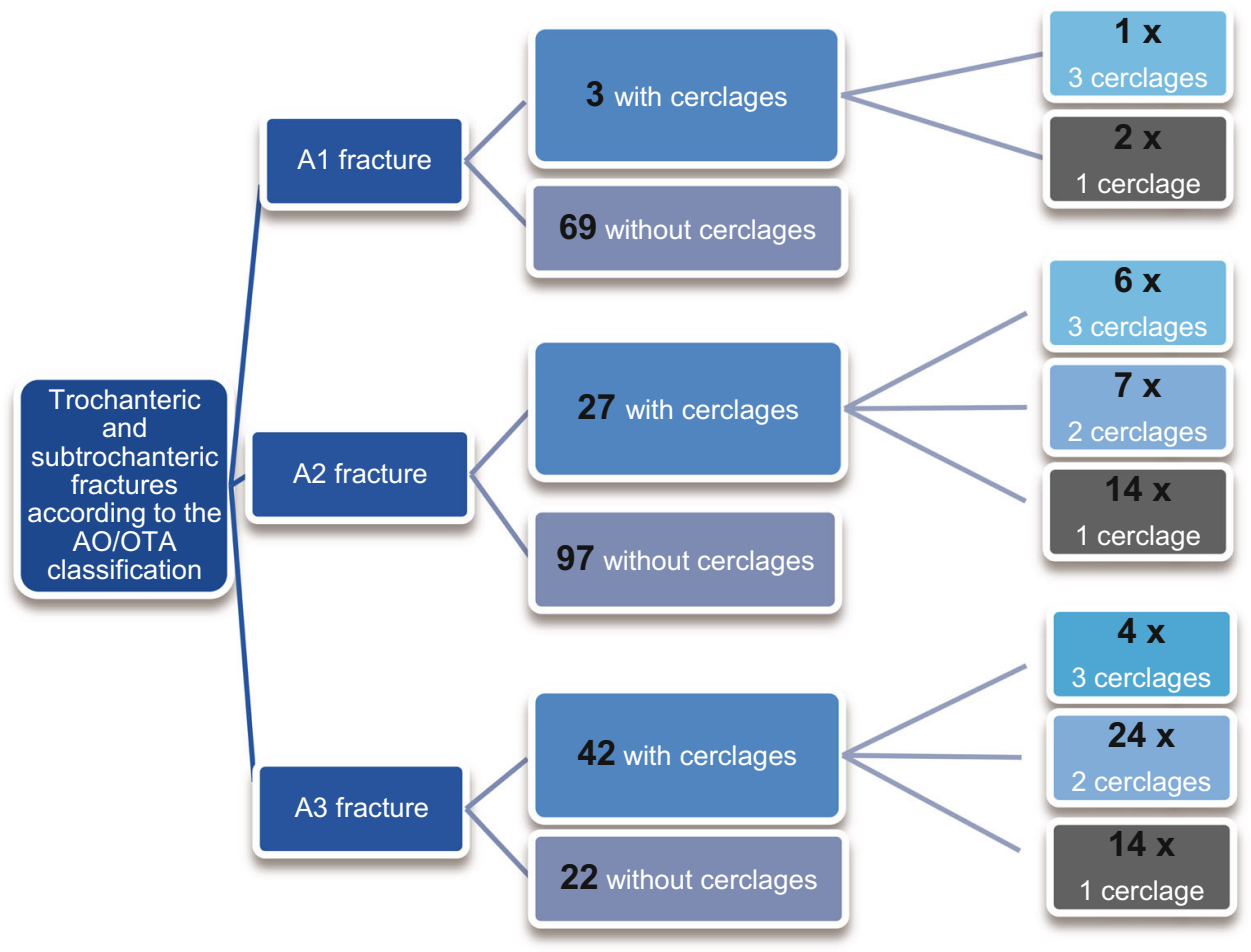
In total, 260 patients were stabilized surgically using a cephalomedullary nail. Among these patients, 72 patients were stabilized with one to three auxiliary cerclages. The distribution to the different fracture configurations is provided in the figure

A retrospective analysis of prospectively collected data from the in-house database in a level I trauma centre was carried out for consecutive patients from January 2014 to March 2019 with AO/OTA 31 A1 to A3 fractures which were stabilized using cephalomedullary nailing with or without the use of additional cable cerclage wiring (Fig. 1). Pre-operative, intra-operative, and post-operative anterior-posterior (AP) and lateral radiographs as well as the entire medical case documentation were examined. In reasoned cases, pre-operatively additional computed-tomography (CT) scans were performed for better visualization of the fracture configuration. Fracture coding according to the AO/OTA classification was performed by two independent observers, both of them senior orthopaedic surgeons.

### Surgical procedure

**Fig. 2:**
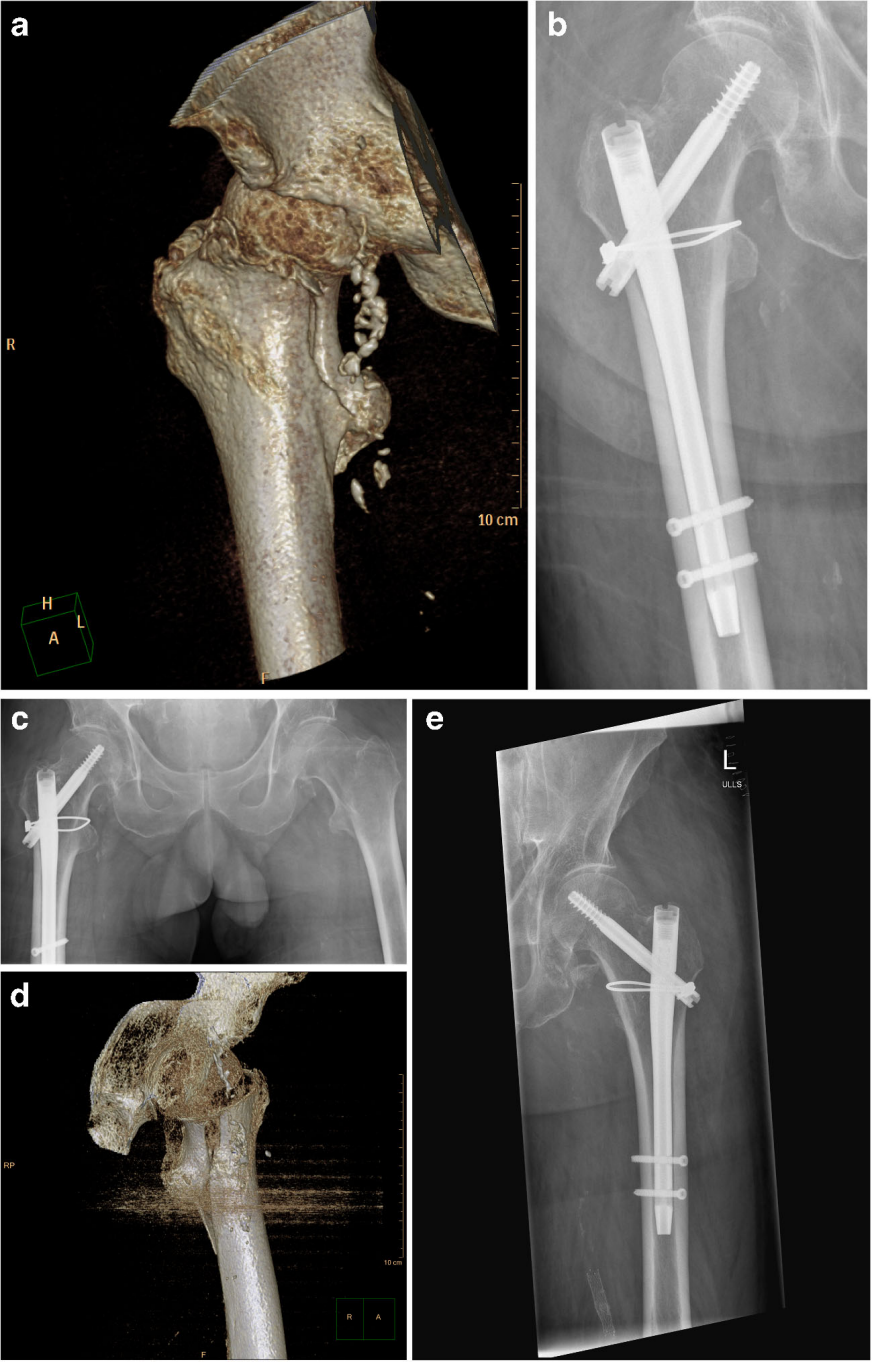
**a** AO/OTA A1.3 trochanteric fracture on the right side in a 72-year-old patient after fall from a standing position. **b** Open reduction and internal fixation (ORIF) was carried out on the traction table using a cephalomedullary nail and one auxiliary cerclage. **c** Three years later, a fall from a standing position in the same patient resulted in an identical AO/OTA A1.3 trochanteric fracture on the left side. Simultaneously, the X-ray provided a 3-year follow-up demonstrating complete fracture healing on the right side. **d** In terms of the complex fracture configuration, an additional CT scan was performed which demonstrated a sagittal fracture line resulting in the decision to use an additional cerclage. **e** Post-operative X-ray demonstrated the situation following ORIF on the traction table with cephalomedullary nail and one additional cerclage

**Fig. 3:**
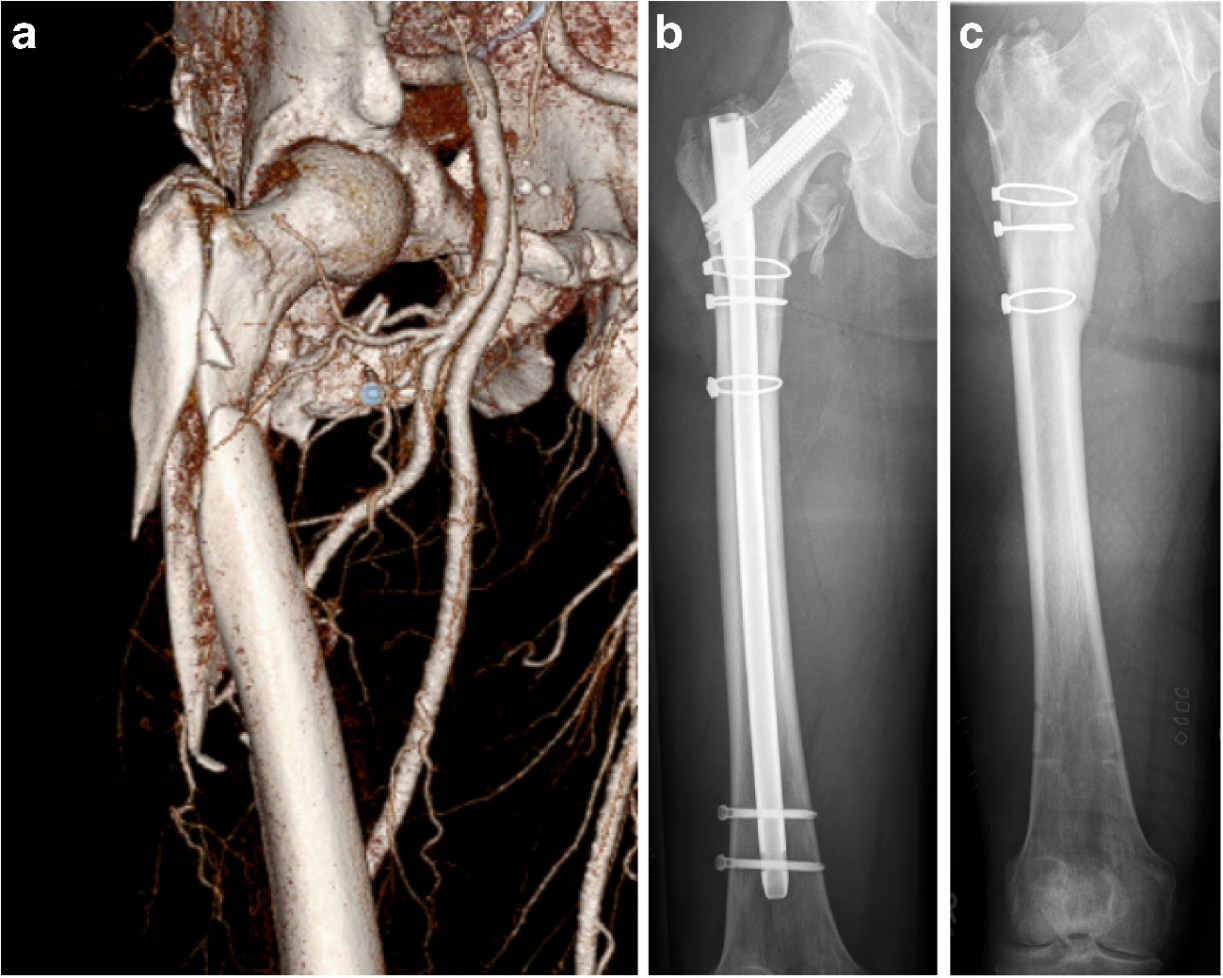
**a** CT scan demonstrating a subtrochanteric AO/OTA A3.3 fracture on the right side in a 62-year-old patient after motorcycle accident. **b** ORIF was performed using a reamed cephalomedullary nail and three auxiliary cerclages with the patient in the lateral decubitus position without any traction device and with free draping of the injured leg. **c** Follow-up 1 year after trauma demonstrating the situation after elective implant removal. The cerclages were left to avoid unnecessary tissue damage

The surgical procedure was carried out in a standardized manner in all cases. Image-controlled storage and reduction was carried out in supine position on the traction table in AO/OTA 31 A1 and A2 fractures (Fig. 2a–e). Fractures were addressed through a standard lateral access and initially reduced in closed technique using the traction table. In AO/OTA 31 A3 fractures, the surgical procedure was performed using the lateral decubitus position without any traction device and free draping of the injured leg [22]. In the group with auxiliary cerclages, open anatomical fracture reduction was performed including the subsequent insertion of one to three 2-mm cerclages (Dall-Miles™ Cable System, Stryker Corp., Kalamazoo, MI, USA: Fig. 3a–c). In both groups, the cephalomedullary nail was implanted according to the manufacturer’s specifications (Gamma3®, Stryker Corp., Kalamazoo, MI, USA; INTERTAN, Smith & Nephew Inc., Memphis, TN, USA). Pathological fractures, periprosthetic fractures, and fractures treated by fracture total hip arthroplasty or by extramedullary fixation devices were excluded from the study, as were patients younger than 18 years and patients who could not give their informed consent.

### Follow-up

Sequential clinical and radiological follow-up examination AP and lateral radiographs were carried out at regular office visits, six weeks, 12 weeks, and at the most recent visit in our outpatient department. The main outcome parameters of the surgical intervention were defined as follows: Patient satisfaction was evaluated using the Lower Extremity Functional Scale (LEFS) [23]. The LEFS consists 20 items, each with a maximum score of 4 points. The total possible score of 80 points indicates a high functional level of the lower extremity. Osseous consolidation rates including stability at the fracture site and radiological elimination of fracture lines in two planes of X-rays were evaluated using the radiographic union score for hip (RUSH) [24]. Besides, complication rates were rated.

### Statistical analysis

The data collected was managed with Excel® for Windows® (Microsoft Corp., Redmond, WA, USA). IBM SPSS® Statistics for Windows 19.0 (IBM Corp., Armonk, NY, USA) was used for statistical evaluation of the results. Results of this study are presented as mean ± standard deviation (SD). The Kolmogorov-Smirnov test was used to check the normal distribution. Significance was statistically calculated based on the *t* test. A result was considered to be statistically significant with *p* value < 0.05.

## Results

The retrospective analysis of our in-house database with regard to trochanteric and subtrochanteric femoral fractures in a five year period revealed a total of 260 patients with AO/OTA A1 to A3 fractures stabilized using a cephalomedullary nail with and without supplemental cerclage wiring. The causes of accident were falls from a standing position in 194 patients, falls from a height more than three metres in 27 patients, high-speed trauma in road traffic in 13 patients, bicycle accidents in 14 patients, and 12 fractures in connection with sports injuries. Of all 260 cases, 256 were primarily presented in our hospital. Twelve fractures occurred in the context of an occupational trauma. One hundred and fifty-five women and 105 men were found among the 260 patients. Mean age of patients was 76.4 ± 15.6 years. Nineteen patients (7%) were younger than 50 years. According to the AO/OTA classification, 72 × A1 fractures (26 × A1.1, 40 × A1.2, 6 × A1.3), 124 × A2 fractures (60 × A2.1, 35 × A2.2, 29 × A2.3), and 64 × A3 fractures (25 × A3.1, 10 × A3.2, 29 × A3.3) were found. In 72 patients, open reduction and internal fixation with cephalomedullary nailing and auxiliary cerclage wiring was used. In these 72 patients with auxiliary cerclages, three A1 fractures, 27 A2 fractures, and 42 A3 fractures were assessed. Among the 72 patients with auxiliary cerclages, six times an intramedullary nail of the standard length (180–200 mm) and 66 times a long intramedullary nail (220–420 mm) were utilized. Eleven times three cerclages (15%), 31 times two cerclages (43%), and 30 times one cerclage (42%) were used (Fig. 1). Operation time was 122.5 ± 36.9 minutes in the treatment group with cerclages versus 66.7 ± 25.6 minutes in the group without cerclages (*p* < 0.001).

Fracture healing according to the Radiographic Union Score for Hip (RUSH) within one year after surgery was evaluated in 248 out of 260 patients (95%). In the patient group without cerclages, osseous consolidation within one year after surgery was found in 180 out of 188 patients (96%). In the patient group with auxiliary cerclages, fracture healing was detected in 68 out of 72 patients (healing rate 94%). The RUSH in the group with cerclages was 28.7 ± 2.2 points and was 28.5 ± 2.2 points in the group without cerclages (*p* = 0.72).

Ninety-one patients were available for a complete functional and radiological follow-up after 38.1 ± 20.4 months in the treatment group with cerclages respectively after 41.9 ± 19.1 months in the treatment group without cerclages (*p* = 0.34), among them 44 females and 47 male patients with an age of 63.6 ± 16.1 years (group with cerclages) versus 65.5 ± 14.5 (group without cerclages) (*p* = 0.56). Fifty-five patients died prior to final follow-up. The remaining patients were lost to follow-up due to relocation and other reasons.

**Fig. 4:**
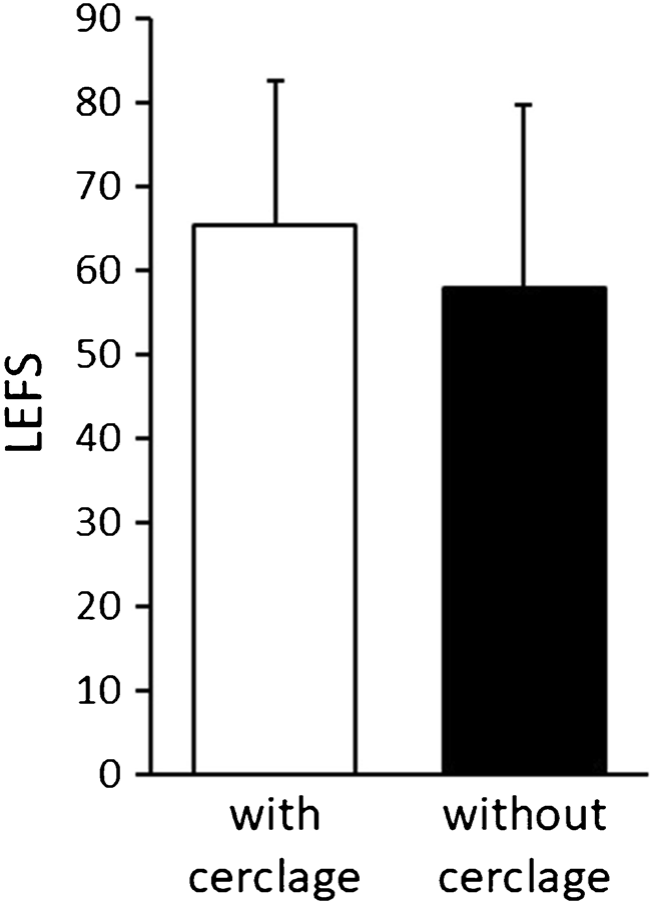
Functional long-term results (mean ± SD) according to the LEFS are significantly better in the treatment group with auxiliary cerclages compared with the group without cerclages (*p* = 0.04). Overall, good functional outcomes were assessed in both treatment groups

Independent of the fracture pattern (A1 + A2 + A3 fractures), functional outcome according to the LEFS was 65.3 ± 17.2 points in the group with cerclages versus 58.4 ± 21 points in the group without cerclages (*p* = 0.04: Fig. 4). The LEFS in AO/OTA type A1 and A2 fractures only was 63.8 ± 17.3 points with auxiliary cerclages versus 62.7 ± 19.3 without cerclages (*p* = 0.84). Focusing on the treatment group with auxiliary cerclages (*n* = 47), the LEFS score was 62.9 ± 17.3 points in AO/OTA type A1 and A2 fractures (*n* = 22) compared with 67.4 ± 15.5 points in AO/OTA type A3 fractures (*n* = 25) (*p* = 0.18).

### Complications

The following intra-operative and post-operative complications with and without use of auxiliary cerclages were determined on the basis of the pre-operative and post-operative radiographs and patients’ data: The complications in the patient group with auxiliary cerclages did not result in any vascular injury, but in three surgical revisions (1 torsional deviation, 1 seroma, and 1 hematoma). Nonunion rate in this treatment group was 6% (4 out of 72 patients). In the patient group without auxiliary cerclages, wound secretion without the need for surgical revision was assessed. Nonunion rate in this group was 4% (8 out of 188 patients).

## Discussion

Although there is still no clear evidence on the superiority of any surgical technique for unstable trochanteric and subtrochanteric fractures, cephalomedullary nailing has been demonstrated to be the most successful treatment option [25]. Despite advances in the surgical management of subtrochanteric and reverse obliquity fractures, in particular with the use of the extended version of cephalomedullary nails, functional outcomes remain worse than in other proximal femoral fractures [26]. Trochanteric fractures possess some aetiological and demographic differences: Women with trochanteric fractures are older, have more severe and generalized bone loss, and more frequently suffer from other osteoporotic fractures [24]. Fracture reduction in elder patients is complicated especially in terms of poor bone quality, comorbidities, and unfavourable fracture configurations [22]. Due to the lack of clear evidence regarding the optimal surgical treatment, implant choice is often based on biomechanical performance: Concerning unstable fractures, modern nail designs including interlocking lag screws provide improved biomechanical performance with controlled impaction of the fracture and a close-to-central weight-bearing axis in the femoral shaft [27]. An optional supplemental cable cerclage is able to minimize the opening of the fracture gap medially and to neutralize relevant varus bending forces in trochanteric and subtrochanteric fractures and thereby maintains the biomechanically important medial pillar providing increased intrinsic primary stability [1, 26]. This supportive effect is located at the heart of any stable osteosynthesis [27]. The medial pillar is under enormous loading pressure when the proximal femur is subjected to axial loads. The lateral pillar, on the other hand, is under the influence of tensile forces [28–31]. If anatomical reduction cannot be achieved sufficiently, the biomechanical basis for the necessary stability and the subsequent bone healing is missing. The supplemental use of cable cerclage wiring also reduces the risk of secondary varization of the axis and related complications [32–35]. The current study can contribute to this key point to the extent that the results have confirmed the above-mentioned intrinsic stability clinically.

Another problem is that common classifications such as the AO/OTA classification do not reasonably represent certain fracture configurations such as sagittal fracture lines (Fig. 2d). Although these fractures then formally correspond to a “simple” fracture shape, in reality, they are much more complex to fix. In these cases, the additional application of an auxiliary cerclage is necessary, although formally, a “simple” fracture according to conventional classifications may be present. Conversely, our results demonstrate that a more complex fracture according to the AO/OTA classification may be present, but in reality, it can be well fixed using singular cephalomedullary nail fixation without the use of additive cerclages.

Although the auxiliary cerclage is a somewhat more invasive additional intra-operative measure, it can in turn reduce the risk of fixation failure relevantly. The damage to the soft tissue has to be weighed against the benefits of the technique. In a previous biomechanical study, it could be demonstrated that after cyclic loading in highly unstable subtrochanteric fractures fixed using cephalomedullary nailing without any additional cerclage, all fractures displaced due to medial dislocation [34]. Conversely, our results encourage the next step to carry out biomechanical tests to measure the exact level of intrinsic stability. Therefore, anatomical reduction of the fracture partners is of great importance for increased healing rates and decreased complication rates [35].

Special care should be taken with regard to vascular injuries when inserting the cerclage. However, if this is standardized and used carefully, the complication rates are known to be relatively low [36]. These findings can be confirmed by the results of our study. After performing a precise surgery protocol, we could not find any significant differences regarding complication rates in both treatment groups.

Another aspect of this study was the operation time. There is common consensus that on open surgical fixation including usage of supplemental fixation tools needs more time than a closed and often called “minimally invasive” reduction technique. In our study, the operation time was significantly longer in the treatment group with cerclages compared with the treatment group without cerclages. But if one takes a look at the fracture configurations treated in both groups, one will find that in the treatment group with cerclages, there are about 60% of AO/OTA type A3 fractures and only about 40% of AO/OTA type A1 and A2 fractures, while in the treatment group without cerclages, only about 10% of the fractures were classified as AO/OTA type A3 fractures (Fig. 1). Hence, the extended operation time is not only with regard to the use of auxiliary cerclages but also to the more complex fracture pattern requiring a more complex surgical technique.

The use of supplemental cerclage wiring is also suspected of disrupting the blood supply to the periosteum and thus endangering bone healing [10–12]. Nevertheless, sufficient evidence for this still is not available. Basically, in contrast to other anatomical regions, the femur seems to be a benign region for the insertion of cerclages [36–39]. We are able to contribute new aspects to this subject in so far that the clinical results of the current study including a healing rate of 95% do not indicate relevant disruption of the blood circulation. Eventually, our clinical results indicate that auxiliary cerclage wiring is more relevantly facilitating anatomical fracture reduction than disrupting the regional blood circulation.

In principle, there is only one relevant study describing that the use of auxiliary cerclages is associated with faster fracture healing, better functional results, and lower complication rates compared with the control group without cerclage [14]. The functional findings of the current study more than three years after surgery demonstrated significantly better functional results after the use of auxiliary cerclages, independent of patients’ age. However, in terms of the fracture healing, we could not find any significant differences between the treatment groups.

Besides, there is also no relevant study concerning the optimal number of cerclages yet. In general, it seems to be important to precisely evaluate the fracture configuration and to classify the fracture according to an established classification system for surgical decision-making. In the present study, one or two cerclages were used in the majority of cases (85%), while three cerclages remained the exception (15%). This is in line with recent studies, where also mainly one cerclage was used [32–35]. In our setting, the use of additional cerclage wiring meanwhile is considered as gold standard with more than 80% of AO/OTA type A3 fractures treated using this technique.

### Study limitations

On the one hand, strengths of this study can be seen in the fact that all patients were managed in the same hospital by the same team of surgeons using a standard treatment protocol provided by Codesido et al. [14]. On the other hand, the retrospective character of the study might be seen as a limitation. It was also not possible to randomize age, gender, and indication for the additional use of cable cerclages, as these were determined by the fracture pattern and by the resulting decision of the treating surgeon and were not subject to a randomized protocol.

## Conclusion

The additional use of cerclages provides intrinsic stability and enables axial alignment and medial cortical support during anatomical fracture reduction and cephalomedullary nail insertion. This contributes to the stability of the entire fixation construct and can maximize the load sharing properties of cephalomedullary nailing resulting in optimal healing rates and minimal complication rates. In the current study, this technique resulted in significantly better functional long-term outcomes than without cerclages. Therefore, it can be recommended as a useful supportive tool especially in comminuted trochanteric and subtrochanteric femoral fractures.

## Data Availability

The datasets analyzed during the current study are available from the corresponding author upon reasonable request.
